# Multiple organ dysfunction caused by a foreign body in the esophagus

**DOI:** 10.5935/0103-507X.20190075

**Published:** 2019

**Authors:** Sasa Dragic, Pedja Kovacevic, Danica Momcicevic, Jovana Cavka, Tijana Kovacevic, Aleksandra Aleksic, Milka Jandric, Biljana Zljutro, Vlado Djajić

**Affiliations:** 1 Medical Intensive Care Unit, University Clinical Centre of the Republic of Srpska - Banja Luka, Republika Srpska, Bósnia e Herzegovina.; 2 Pan-European University "Apeiron" - Banja Luka, Bósnia e Herzegovina.; 3 Medical School, University of Banja Luka - Banja Luka, Bósnia e Herzegovina.; 4 Clinical Pharmacy, University Clinical Centre of the Republic of Srpska - Banja Luka, Bósnia e Herzegovina.; 5 Department of Ear, Throat, and Nose Diseases, University Clinical Centre of the Republic of Srpska - Banja Luka, Bósnia e Herzegovina.; 6 University Clinical Centre of the Republic of Srpska - Banja Luka, Bósnia e Herzegovina.

**Keywords:** Foreign bodies, Multiple organ failure, Mediastinitis

## Abstract

We present the case of a 71-year-old patient who was admitted to the medical intensive care unit in a state of multiple organ dysfunction. After the fourth day of applying all needed life-saving measures (vasopressor stimulation, mechanical ventilation, continuous dialysis treatment, broad spectrum antibiotic therapy, and other supportive measures), nonspecific heteroanamnestic data revealed that the patient had been having a persistent difficulty in swallowing liquids and food for a few days prior to hospital admission. After performing additional radiological and endoscopic diagnostic procedures, a foreign body was detected; a steel wire that had a length of approximately 6cm and was bent in a half had penetrated the esophagus and was projected into the seventh neckline. We managed to evacuate the foreign body endoscopically without further complications, and we stabilized our patient using additional therapeutic measures as needed.

## INTRODUCTION

In the adult population, foreign body ingestion is usually seen in alcohol abusers, psychotropic drug users, and patients with mental disorders or bulbar symptomatology.^(1)^ Etiologically, prosthetic material and pieces of food that were not or could not be adequately chewed appear most common (meat, bone, teeth).^(2)^ The clinical presentation is usually very dramatic and immediately recognizable, and anamnestic or heteroanamnestic data directs a diagnostic approach. According to different studies, the frequency of foreign bodies in the digestive system is between 28 and 68%. The most common symptoms associated with foreign body ingestion are dysphagia, odynophagia, vomiting, retrosternal pain, accumulation of saliva in the mouth and respiratory tract symptoms.^(3)^ Foreign bodies are most commonly found in one of three narrowings of the esophagus: the cricopharyngeal muscle, at the aortic pressure level or at the cardia.^(4)^ X ray and CT (computed tomography) are believed to be the most helpful diagnostic procedures for foreign body detection. CT angiography is considered when there is suspicion of foreign body migration to nervous tissue, while MRI is contraindicated when a metal structure of the foreign body is suspected.^(5)^ Removal of the foreign body is crucial for the prevention of infections and life-threatening complications such as perforation and chemical mediastinitis. There are few therapeutic options available for the management of foreign body ingestion, and the choice depends on the patient's age, clinical presentation, and the size, shape and location of the foreign body. Endoscopy is the most often used method with the lowest mortality rate. It must be taken into account that some foreign bodies cannot be evacuated using endoscopy, and complications can occur, such as damage to surrounding tissues or foreign body migration, after which surgery is the only management option.^(6)^ Foreign bodies usually migrate into surrounding tissues, and migration it is not always be caused by an attempted manipulation or removal; it can cause infection, abscess or necrotizing mediastinitis, which are very serious complications associated with a high mortality rate.^(7-9)^

## CASE REPORT

A seventy-one-year-old patient was admitted to the medical intensive care unit, University Clinical Centre of Republic of Srpska (MICU), after one day of treatment in the internal medicine clinic for an acute kidney disorder with extreme metabolic acidosis, impaired consciousness, hemodynamic instability and signs of sepsis.

Fourteen days prior to current hospitalization, the patient was examined by an internist for scanty urination and elevated parameters of inflammation: C reactive protein (CRP) 210.8mg/L (ref range: 0 - 5mg/L) and *white blood cells* (WBC) 12.5 x 10^9^/L (reference range: 3,40 - 9,70 x 10^9^/L) with a serum urea level of 13.2mmol/L (reference range: 2.8 - 7.2mmol/L) and serum creatinine level of 147mmol/L (reference range: 45 - 84mmol/L). The internist prescribed antibiotic treatment for ten days after which the patient felt better. During a scheduled follow-up visit with the internist, an ultrasound of the kidneys and abdomen was performed and showed no abnormalities; urea and creatinine in the serum were 18.1mmol/L and 226mmol/L, respectively. The patient had diabetes and myocardial infarction in her medical history.

The patients' laboratory parameter levels recorded immediately after admission to the MICU were as follows: urea 36.6mmol/L (reference range: 2.8 - 7.2mmol/L), creatinine 1297mmol/L (reference range: 45 - 84mmol/L); potassium 7.5mmol/L (reference range: 3.5 - 5.1mmol/L); and WBC 15.39x10^9^/L (3,40 - 9,70 x 10^9^/L). Arterial blood gas analysis showed pH 6.909 (reference range: 7.34 - 7.45); *partial pressure of carbon dioxide* (pCO_2_) 2.82kPa (reference range: 4.27 - 6.0 kPa); *partial pressure of oxygen* (pO_2_) 8.7kPa (reference range: 11.07 - 14.40kPa); oxygen *saturation* (SatO_2_) 83% (reference range: 95 - 99%), bicarbonate (HCO_3_^-^) 4mmol/L (reference range: 21.2 - 27mmol/L); and base excess (BE) -27.7.

Life-saving procedures were started on admission, including intubation and controlled mechanical ventilation, continuous analgosedation, urgent dialysis, fluid resuscitation, vasopressor stimulation, empirical wide-spectrum antibiotics, corticosteroid therapy and other supportive and symptomatic treatments.

**Figure 1:**
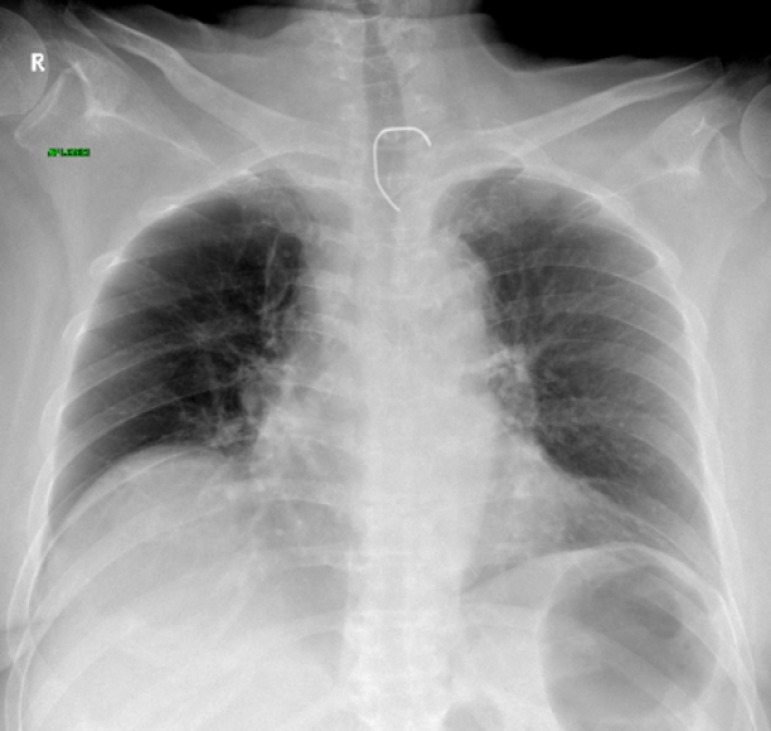
Chest X-ray showing foreign body.

**Figure 2:**
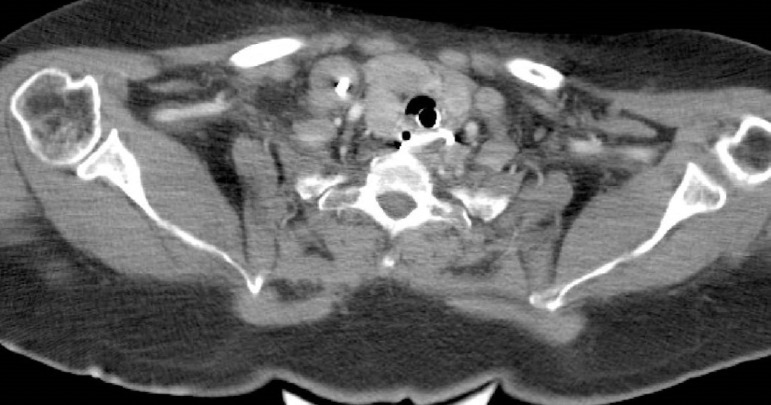
Computed tomography of the neck show a prevertebral shadow of an object with the density of a metal object.

Due to persistent metabolic acidosis and profound hemodynamic instability, continuous venovenous hemodiafiltration was initiated and continued until the patient's metabolic status was stabilized. The initial radiological examination revealed a foreign body that had the density of a metal object (chest X ray) (Figure 1), but it was not detected in profile shots. Therefore, a CT of the patients' neck was performed and showed a prevertebral shadow of an object with the density of a metal object (outside of the esophageal lumen) at the C7-Th 1 level, but the origin of the object could not be verified (Figure 2).

The patient reached a more stabilized metabolic condition in the next few days, and she was weaned from the vasopressors and mechanical ventilators. Taking a closer look at the patient's condition during conversations with her family, it became known that a few days prior to the appearance of first symptoms, the patient felt a disturbance after having lunch (pork meets from a barbeque (BBQ), which prevented her from eating and drinking normally and resulted in an elevation in serum urea and creatinine with decreased urine output and elevation in inflammation parameters.

The radiological examination was immediately repeated, and with the cooperation of an otorhinolaryngology specialist, esophagoscopy with a rigid instrument was performed under general endotracheal anesthesia. A foreign body was detected (a steel wire that is most likely part of the steel wire that was used to attach the BBQ meat to the skewer while baking) in the esophageal lumen, 20cm from the maxillary alveolar ridge with the right end of the wire passing through the lumen on its side and back; the esophageal mucosa was edematous, pale and covered with fibrin deposits.

**Figure 3:**
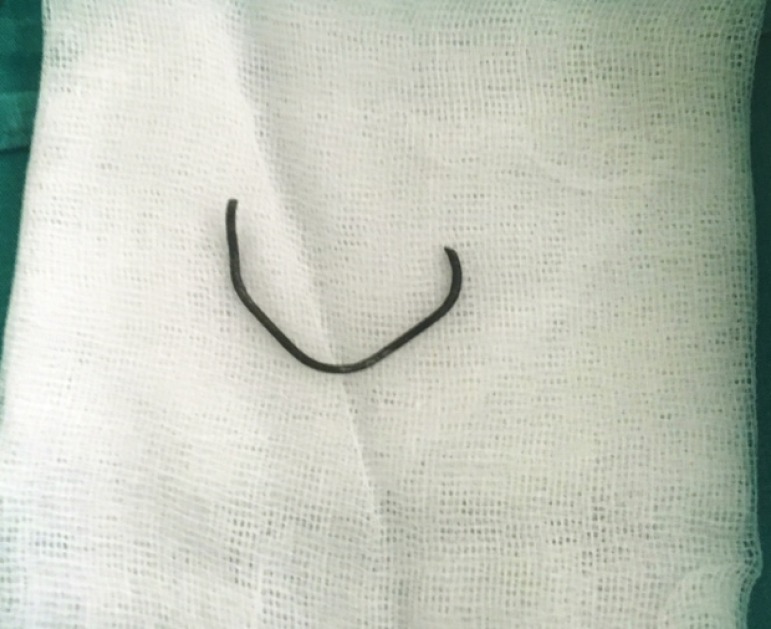
Foreign body.

After orientation, a metal wire with a length of 6cm was completely removed from the esophageal lumen (Figure 3). Further treatment included empirical antimicrobial coverage for possible mediastinitis infection due to the perforation injury of the esophagus. After the procedure, the patient was clinically stable with for the intention of full recovery, and she was transferred to a step-down unit.

## DISCUSSION

Taking into account all the events described, we can conclude that the described case was not specific on more bases. The symptom for which the patient was examined by the family physician and later by the internal medicine specialist was decreased urine output, which was interpreted as a sign of diabetic nephropathy. Rapid deterioration in renal function, sepsis and multiorgan dysfunction required urgent actions from a critical care physician in terms of the provision of life support regardless of true etiological cause, which was determined later by accidental additional heteroanamnestic reconstruction.

The long latency period contributed to minimizing the initial dysphagia and discomfort. The diagnostic approach in this case identified the possible presence of a foreign body, but precise localization (luminal or extraluminal) was not possible. Due to the long experience of the otorhinolaryngologist in endoscopic procedures, the differential diagnosis was confirmed, and a detailed evaluation of the position of sharp parts of the foreign metal object was made, which resulted in its evacuation without additional complications.

## CONCLUSION

Despite all available advanced diagnostic procedures, anamnestic/heteroanamnestic data are sometimes crucial in the diagnosis or identification of the etiological factor. In this case report, the symptoms did not indicate the presence of a foreign body in the esophagus, but with further reconstruction of the events, a causal relationship between those two appeared logical and possible.
